# Erratum: Kim, J.; et al. Characterization of *mcr-1*-Harboring Plasmids from Pan Drug-Resistant *Escherichia coli* Strains Isolated from Retail Raw Chicken in South Korea. *Microorganisms* 2019, *7*, 344

**DOI:** 10.3390/microorganisms7100470

**Published:** 2019-10-18

**Authors:** Jinshil Kim, Bo Kyoung Hwang, HyeLim Choi, Yang Wang, Sang Ho Choi, Sangryeol Ryu, Byeonghwa Jeon

**Affiliations:** 1 Department of Food and Animal Biotechnology, Research Institute for Agriculture and Life Sciences, Center for Food and Bioconvergence, Seoul National University, Seoul 08826, Korea; jinsilk1130@naver.com; 2 Department of Agricultural Biotechnology, Seoul National University, Seoul 08826, Korea; 3 Department of Agricultural Biotechnology, Center for Food Safety and Toxicology, Seoul National University, Seoul 08826, Korea; rose0814p@naver.com (B.K.H.); helenchoi501@gmail.com (H.C.); choish@snu.ac.kr (S.H.C.); 4 Food-Borne Pathogen Omics Research Center (FORC), Seoul National University, Seoul 08826, Korea; 5 Beijing Advanced Innovation Center for Food Nutrition and Human Health, College of Veterinary Medicine, China Agricultural University, Beijing 100083, China; wangyang@cau.edu.cn; 6 Environmental Health Sciences, School of Public Health, University of Minnesota, Minneapolis, MN 55455, USA

The authors would like to make the following corrections to the published paper [1].

We would like to change the affiliation on Page 1 of paper [1] from

Jinshil Kim ^1^, Bo Kyoung Hwang ^2,3^, HyeLim Choi ^2,3^, Yang Wang ^4^, Sang Ho Choi ^2,3^, Sangryeol Ryu ^1^^,^* and Byeonghwa Jeon ^1,5,^*

^1^  Department of Food and Animal Biotechnology, Research Institute for Agriculture and Life Sciences, Center for Food and Bioconvergence, Seoul National University, Seoul 08826, Korea; jinsilk1130@naver.com
^2^  Department of Agricultural Biotechnology, Center for Food Safety and Toxicology, Seoul National University, Seoul 08826, Korea; rose0814p@naver.com (B.K.H.); helenchoi501@gmail.com (H.C.); choish@snu.ac.kr (S.H.C.)
^3^  Food-borne Pathogen Omics Research Center (FORC), Seoul National University, Seoul 08826, Korea
^4^  Beijing Advanced Innovation Center for Food Nutrition and Human Health, College of Veterinary Medicine, China Agricultural University, Beijing 100083, China; wangyang@cau.edu.cn
^5^  Environmental Health Sciences, School of Public Health, University of Minnesota, Minneapolis, MN 55455, USA

∗  Correspondence: sangryu@snu.ac.kr (S.R.); bjeon@umn.edu (B.J.)

to the correct version as follows:

Jinshil Kim ^1,2^, Bo Kyoung Hwang ^3,4^, HyeLim Choi ^3,4^, Yang Wang ^5^, Sang Ho Choi ^3,4^, Sangryeol Ryu ^1^^,2,^* and Byeonghwa Jeon^1,2,6,^*

^1^  Department of Food and Animal Biotechnology, Research Institute for Agriculture and Life Sciences, Center for Food and Bioconvergence, Seoul National University, Seoul 08826, Korea; jinsilk1130@naver.com
^2^  Department of Agricultural Biotechnology, Seoul National University, Seoul 08826, Korea
^3^  Department of Agricultural Biotechnology, Center for Food Safety and Toxicology, Seoul National University, Seoul 08826, Korea; rose0814p@naver.com (B.K.H.); helenchoi501@gmail.com (H.C.); choish@snu.ac.kr (S.H.C.)
^4^  Food-borne Pathogen Omics Research Center (FORC), Seoul National University, Seoul 08826, Korea
^5^  Beijing Advanced Innovation Center for Food Nutrition and Human Health, College of Veterinary Medicine, China Agricultural University, Beijing 100083, China; wangyang@cau.edu.cn
^6^  Environmental Health Sciences, School of Public Health, University of Minnesota, Minneapolis, MN 55455, USA

∗  Correspondence: sangryu@snu.ac.kr (S.R.); bjeon@umn.edu (B.J.)

The authors would like to apologize for any inconvenience caused to the readers by these changes. The changes do not affect the scientific results. The manuscript will be updated and the original will remain online on the article webpage.
