# Supplementary material for: Characterization of mcr-1-Harboring Plasmids from Pan Drug-Resistant Escherichia coli Strains Isolated from Retail Raw Chicken in South Korea
Source: Microorganisms. 2019 Sep 12;7(9):344. doi: 10.3390/microorganisms7090344 (PMC6780365; doi:10.3390/microorganisms7090344)
Supplement: Supplementary file 1 [file microorganisms-07-00344-s001.pdf]

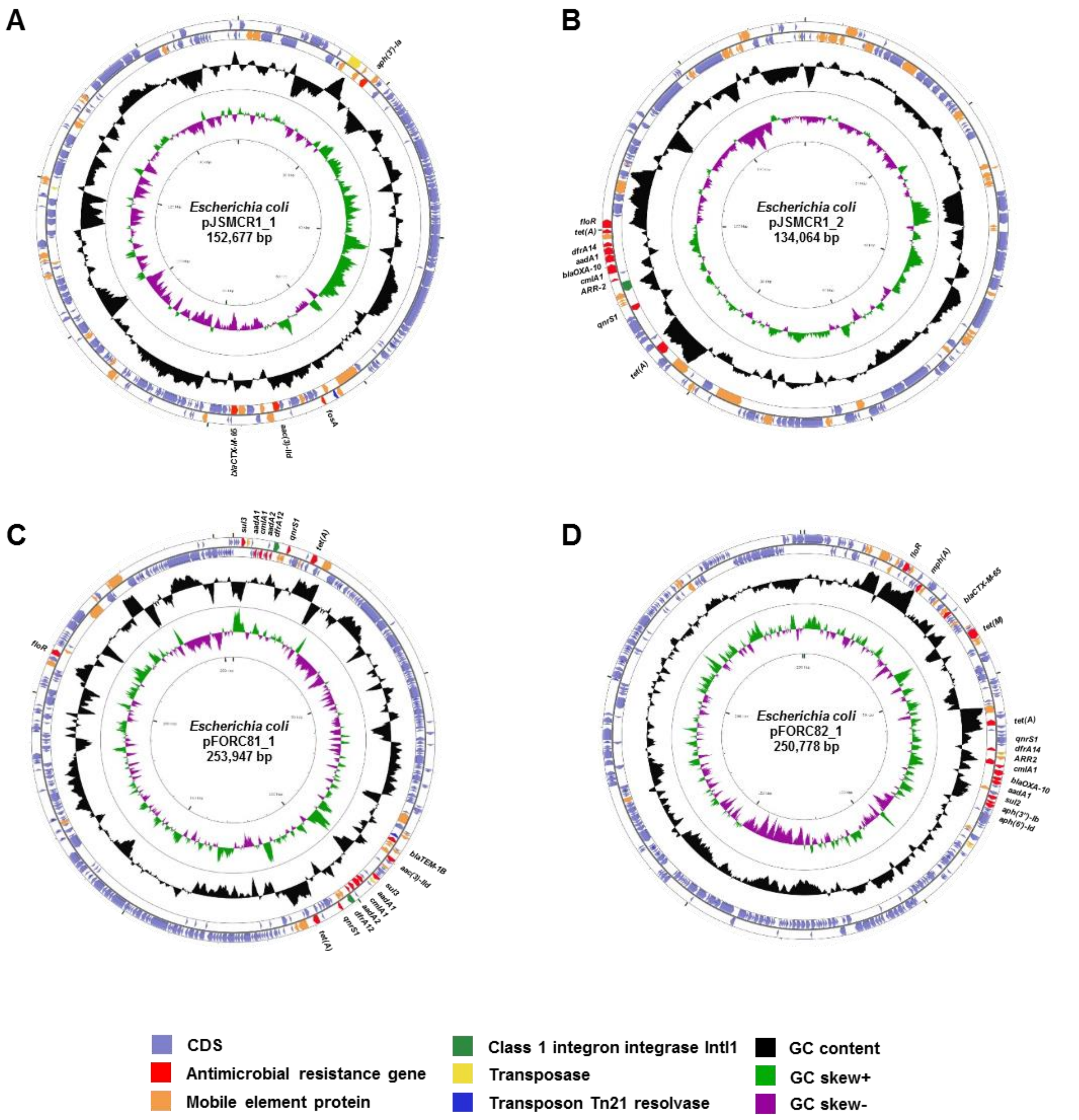

Fig. S1. Circular map of plasmid pJSMCR1_1 (A), pJSMCR1_2 (B), pFORC81_1 (C), and pFORC82_1 (D). Functions are color coded as explained in the key. GC content and GC Skew are represented on the distance scale (in Kbp) on the inner map.
